# Evaluating artificial intelligence-generated clinical guidance for patellar dislocation: accuracy, readability and qualitative appraisal of DeepSeek-R1 responses

**DOI:** 10.3389/fdgth.2026.1719101

**Published:** 2026-09-29

**Authors:** Shuo Liu, Xiyang Sun, Yunfei Ma, Zhenzhong Yang, Hanyuan Zhang, Hongbo Mao, Bin Xu

**Affiliations:** 1 Department of Orthopedics, Department of Sports Medicine and Arthroscopic Surgery, The First Affiliated Hospital of Anhui Medical University, Hefei, China; 2 First School of Clinical Medicine, Anhui Medical University, Hefei, China; 3 Department of Orthopedics, Department of Sports Medicine and Arthroscopic Surgery, The Fourth People's Hospital of Lu'an, Lu'an, China

**Keywords:** artificial intelligence (AI), ChatGPT, clinical decision support, DeepSeek, large language model (LLM), patellar dislocation

## Abstract

**Introduction:**

Large language models (LLMs) are increasingly used to answer patient questions, but the readability and information quality of patellar dislocation guidance remain unclear.

**Methods:**

A series of clinical questions covering etiopathogenesis, mechanisms, clinical manifestations, diagnosis, treatment, complications, and postoperative rehabilitation of patellar dislocation were posed to DeepSeek-R1 v2.3.1 and ChatGPT-4o. Readability was assessed using the Flesch Reading Ease score (FRES), Flesch-Kincaid Grade Level (FKGL), Automated Readability Index (ARI), SMOG Index, and Gunning Fog Score (GFS). Three sports medicine surgeons rated responses using DISCERN. Wilcoxon signed-rank tests compared models; Inter-rater reliability (IRR) for DISCERN was assessed with intraclass correlation coefficient (ICC). Additionally, we analysed DeepSeek's responses based on the current literature and therapeutic standards.

**Results:**

Readability favored ChatGPT-4o across all indices (between-model *P* < 0.05): DeepSeek-R1 vs. ChatGPT-4o medians (Q1–Q3) were ARI 15.62 (13.90–16.60) vs. 12.54 (10.15–13.41), FKGL 14.39 (12.38–15.24) vs. 11.27 (9.44–12.76), GFS 18.25 (15.20–19.43) vs. 14.55 (13.73–16.53), SMOG 11.02 (9.72–12.18) vs. 9.55 (8.14–10.67), and FRES 9.50 (6.75–25.00) vs. 33.00 (19.75–41.50). Both models exceeded the NIH-recommended sixth-grade reading level (all *P* < 0.05). DISCERN scores were similar [median (Q1–Q3)] for DeepSeek-R1 vs. ChatGPT-4o: 49.17 (40.25–59.17) vs. 50.33 (38.92–58.42), corresponding to “Fair” quality (*P* = 0.767). DISCERN agreement was high (single-measure ICC 0.860–0.950).

**Conclusions:**

Although both AI platforms can generate clinically relevant responses of moderate quality, they should be regarded as adjunctive information rather than stand-alone clinical guidance, particularly given the high incidence of patellar instability and developmental characteristics of children and adolescents. Exploratory qualitative assessment of DeepSeek-R1 responses identified strengths in clinical explanation and reasoning, while also revealing limitations in citation reliability and readability. Future work should incorporate multi-turn, multi-generation frameworks, more samples with repeated sampling and longitudinal assessment, enforced citation standards, and expanded evaluation to better quantify both clinical utility and reliability.

## 1 Introduction

Patellar dislocation is a common knee disorder. Among children and adolescents, the incidence of first-time patellar dislocation is high (42/100,000), with the highest rate observed in female patients aged 10–17 years (108/100,000). The average risk of recurrent dislocation after the first dislocation is 22.7%, rising to 35.5% in the 10–17-year age group (1). Patellar dislocation can substantially impair knee function, activity level, and quality of life. In addition to traumatic events, several predisposing factors—such as patella alta, trochlear dysplasia, generalized ligamentous laxity, and an increased tibial tuberosity–trochlear groove (TT–TG) distance—may increase the risk of recurrent instability (2).

With the advancement of technology, channels for accessing information have become increasingly diverse and convenient. Beyond browsing videos, websites, and search engine queries, AI-powered chat has emerged as a novel approach to obtaining information, demonstrating significant potential across various fields such as science, technology, and healthcare. According to a report from November 20, 2025, ChatGPT reportedly has ∼800 million weekly active users, up from ∼400 million in February 2025 (Singh S. ChatGPT users stats (December 2025) - growth & usage data. DemandSage. November 20, 2025. Accessed December 9, 2025. https://www.demandsage.com/chatgpt-statistics/). AI can retrieve information from vast online databases and provide tailored responses, while continuous dialogue enables more personalized answers, better meeting the needs of users. Building on this, many physicians have proposed leveraging AI to advance the medical field, primarily focusing on three critical domains: augmenting medical education through case-based reasoning simulations, enhancing clinician workflow efficiency, and addressing various patient needs via adaptive communication interfaces (3).

Multiple empirical studies have systematically evaluated AI dialogue systems using patient-centric medical inquiries. Several investigations have demonstrated that advanced chatbots, such as ChatGPT-4, not only perform competitively on standardized medical licensing examinations but also generate clinically appropriate responses with reasonable professional accuracy and readability (4–14). However, some relevant studies have also indicated that AI may not provide satisfactory responses to medical-related inquiries, with some answers exhibiting poor readability that makes them difficult for users to understand (15).

Current literature reveals a paucity of studies evaluating AI chatbots in orthopedic subspecialties, with most existing research focusing predominantly on ChatGPT-4 applications (4, 5, 9, 11, 14–16). Within orthopedics, as patellar dislocation disproportionately affects adolescents, AI-generated educational content must appropriately address age-specific considerations, including skeletal maturity and differences in surgical decision-making. To further explore the capabilities and limitations of emerging AI chatbots in orthopaedic patient education, we focus on DeepSeek, a new AI chat model launched in 2025. This study centers on a novel LLM—DeepSeek-R1 v2.3.1, comparing it with ChatGPT-4o. Our study centers on DeepSeek-R1 v2.3.1 and compares its performance with ChatGPT-4o across two critical dimensions: professional accuracy and readability, while exploring potential and limitations in medical information retrieval. Qualitative findings are interpreted as exploratory observations rather than direct comparative evidence.

## 2 Methods

### 2.1 Ethical approval

This study did not include human subject research, and it did not require “Ethics Committee Approval”.

### 2.2 Question design

The formulation of the questions was guided by a modified Delphi consensus statement (17). An associate chief orthopedic surgeon summarized and refined ten core clinical questions covering the full continuum of patellar dislocation care—including etiology, mechanisms, diagnosis, treatment, and rehabilitation—to enable detailed expert evaluation of each response, along with qualitative content analysis. Given that this study analyzes each AI's response and its characteristics, we prioritized depth of assessment over a larger number of answers.

### 2.3 AI types, models and generation settings

Data collection was performed on April 24, 2025. The DeepSeek-R1 v2.3.1 and ChatGPT-4o versions available through their official interfaces at that time were evaluated. Our study used the publicly available DeepSeek-R1 (v2.3.1) with “DeepThink” and “Search” turned on (simulating the real usage experience of users), other parameters were left at the platform defaults: the maximum generation length was 32,768 tokens, with a temperature of 0.6 and a top-*p* of 0.95. For ChatGPT-4o, responses were generated using the default settings available in the user interface at the time of data collection. The model supports a maximum context window of 128,000 tokens, with a temperature of 0.7 and a top-*p* of 0.9.

Differences in decoding parameters may influence response diversity and wording complexity. However, because these parameters were not independently adjustable in both platforms, complete parameter harmonization was not feasible. Our study reflected the publicly available default user experience at the time of data collection. Therefore, our comparison should be interpreted as a comparison of real-world accessible outputs rather than strictly controlled model performance.

To reduce response variability and improve comparability across models, we implemented a standardized querying and data collection workflow to minimize the influence of personalization settings and contextual factors on LLM outputs. Specifically, both DeepSeek-R1 and ChatGPT-4o were queried under identical conditions: memory and custom instructions were disabled before data collection. The chat history was cleared before entering a new question; once the chatbot provided its response, the session history was deleted, and no follow-up messages were sent thereafter.

### 2.4 Readability and quality assessment

Readability analysis was performed using five established metrics: the Flesch Reading Ease score, Flesch-Kincaid Grade Level, Automated Readability Index, SMOG Index, and Gunning Fog Score (18–21). The FRES evaluates public-facing content on a scale from 0 to 100, with higher scores indicating greater ease of comprehension. The FKGL, a derivative of the FRES, estimates the U.S. grade level required to understand the text. The GFS measures the proportion of complex words, while the SMOG Index is particularly suitable for assessing the readability of health education materials. All readability scores were calculated using the Readability Scoring System (Readability Scoring System PLUS with the Robert Gunning Editor). As patellar dislocation disproportionately affects children and adolescents, the results of this study were compared against the sixth-grade reading level recommended by the American Medical Association and the National Institutes of Health (NIH). An average acceptable score of 80.00 is used for the FRES, and a score of 6.00 is used for the other four formulas (22).

We applied the DISCERN instrument, which enables patients, clinicians, and researchers to assess the quality of written health information. The tool consists of 16 questions divided into two sections: Section 1 (Q1–Q8) assesses the reliability of the publication, and Section 2 (Q9–Q15) evaluates the quality of information on treatment options; Question 16 provides an overall quality rating. Each item is scored on a 5-point scale (1–5), and the total score (range 16–80) can be categorized into five quality levels: ≤26 (very poor), 27–38 (poor), 39–50 (fair), 51–61 (good), and ≥62 (excellent) (23, 24).

Three trained sports medicine surgeons independently assessed all AI responses using the DISCERN instrument. They first conducted a pilot assessment of three other texts. Subsequently, the three surgeons discussed and reached consensus on the scoring criteria, after which they independently evaluated the AI-generated responses. IRR was evaluated using the ICC (25, 26). Given DeepSeek's novelty, we conducted an in-depth qualitative appraisal of DeepSeek-R1 responses; full responses for both models are provided in the Supplementary Materials.

### 2.5 Statistical analysis

Continuous data are expressed as the median (interquartile range: Q1–Q3). Readability outcomes (Flesch Reading Ease, Flesch–Kincaid Grade Level, Automated Readability Index, SMOG Index, and Gunning Fog Score) and DISCERN scores were treated as continuous variables. Given the small paired sample size (*n* = 10) and the presence of ordinal outcomes, between-model comparisons for all results were conducted using the Wilcoxon signed-rank test for related samples. Also, Wilcoxon signed-rank tests were used to compare each readability metric against the sixth-grade benchmark. Given the small paired sample size (*n* = 10), it is underpowered to detect small between-model differences. Therefore, non-significant *p*-values should not be interpreted as evidence of equivalence. A two-way random-effects model with absolute agreement of ICC was applied to quantify agreement among the three raters. Both single-measure ICC and average-measure ICC (based on the mean of three raters) were reported with 95% confidence intervals. Statistical analyses were performed using IBM SPSS Statistics, version 24.0. *P* value < 0.05 was considered statistically significant (Figure 1).

**Figure 1:**
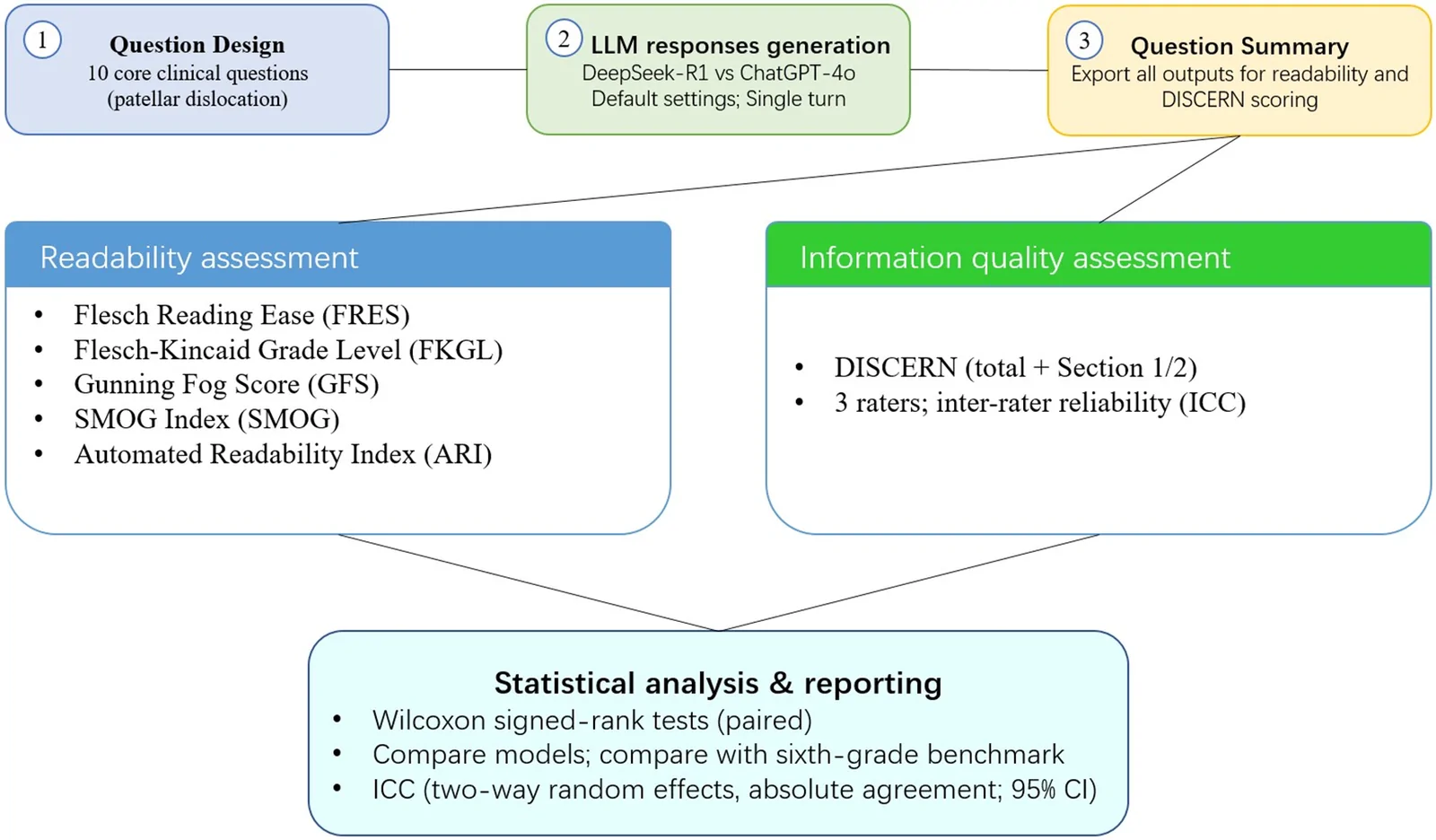
Study workflow for evaluating LLM responses generation on patellar dislocation.

## 3 Results

Details are presented in the Supplementary Material, including the responses from ChatGPT-4o (Supplementary Material S1) and DeepSeek-R1 (Supplementary Material S2).

We collected responses generated by DeepSeek-R1 and ChatGPT-4o, computed five readability scores for each response, and conducted between-model comparisons. We compared the readability of each AI-generated text with the readability recommendations of the American Medical Association and the National Institutes of Health. The results are summarized in Table 1.

**Table 1:** Readability scores for DeepSeek-R1 and ChatGPT-4o responses to the patellar dislocation-related questions.

Statistics	DeepSeek-R1	Chat-GPT4o	Chat-GPT C6thGRL (P)*^,^^a^	DeepSeek C6thGRL (P)*^,^^a^	Between ChatGPT and DeepSeek (P)^a^
ARI	15.62 (13.90–16.60)	12.54 (10.15–13.41)	<0.01	<0.01	<0.01
FRES	9.50 (6.75–25.00)	33.00 (19.75–41.50)	<0.01	<0.01	<0.01
GFS	18.25 (15.20–19.43)	14.55 (13.73–16.53)	<0.01	<0.01	=0.01
FKGL	14.39 (12.38–15.24)	11.27 (9.44–12.76)	<0.01	<0.01	<0.01
SMOG	11.02 (9.72–12.18)	9.55 (8.14–10.67)	<0.01	<0.01	=0.02

Results are summarized as median (Q1–Q3).

*p* values in bold are statistically significant.

* C6thGRL (p): Comparison of the responses according to 6th grade reading level (p).

a Wilcoxon test.

ARI, automated readability index; FKRS, flesch reading ease score; GFS, gunning fog score; FKGL, flesch-kincaid grade level; SMOG, simple measure of gobbledygook.

Readability favored ChatGPT-4o across all indices (between-model *P* < 0.05): DeepSeek-R1 vs. ChatGPT-4o medians (Q1–Q3) were ARI 15.62 (13.90–16.60) vs. 12.54 (10.15–13.41), FKGL 14.39 (12.38–15.24) vs. 11.27 (9.44–12.76), GFS 18.25 (15.20–19.43) vs. 14.55 (13.73–16.53), SMOG 11.02 (9.72–12.18) vs. 9.55 (8.14–10.67), and FRES 9.50 (6.75–25.00) vs. 33.00 (19.75–41.50). Both models showed significantly poorer readability than the NIH-recommended sixth-grade reading level (*P* < 0.05; Table 1).

Table 2 Shows the DISCERN and each section of DISCERN's analysis.

**Table 2:** DISCERN scores for DeepSeek-R1 and ChatGPT-4o responses to the patellar dislocation-related questions.

Statistics	DeepSeek-R1	Chat-GPT4o	Between ChatGPT and DeepSeek (P)^a^
DISCERN	49.17 (40.25–59.17)	50.33 (38.92–58.42)	= 0.767
DISCERN-S1	27.33 (26.58–30.75)	27.00 (26.25–30.17)	= 0.165
DISCERN-S2	19.00 (8.75–24.08)	19.00 (7.67–22.42)	= 1.00

Results are summarized as median (Q1-Q3).

No *p* value is significant.

a Wilcoxon test.

DOSCERN-S1: First part of DISCERN, assessing the reliability of the publication; DISCERN-S2: Second part of DISCERN, evaluates the quality of information on treatment options.

For quality outcomes, both DeepSeek-R1 and ChatGPT-4o achieved “Fair” overall quality as measured by DISCERN, with median (IQR) total scores of 49.17 (40.25–59.17) and 50.33 (38.92–58.42), respectively. For these two models, DeepSeek-R1 and ChatGPT-4o obtained scores of DISCERN-S1 [27.33 (26.58–30.75) vs. 27.00 (26.25–30.17)] and DISCERN-S2 [19.00 (8.75–24.08) vs. 19.00 (7.67–22.42)]. No statistically significant between-model difference was detected for DISCERN total score (*P* = 0.767), DISCERN-S1 (*P* = 0.165) or DISCERN-S2 (*P* = 1.00). However, the small sample size limits the statistical power to detect potentially meaningful differences.

Regarding the ICC analysis, agreement among the three raters was high for DISCERN total scores in both models. For ChatGPT-4o responses (*n* = 10), the single-measure ICC was 0.860 (95% CI, 0.658–0.959) and the average-measure ICC was 0.948 (95% CI, 0.852–0.986; *P* < 0.001). For DeepSeek-R1 responses (*n* = 10), the single-measure ICC was 0.950 (95% CI, 0.829–0.987) and the average-measure ICC was 0.983 (95% CI, 0.936–0.996; *P* < 0.001).

Given that DeepSeek-R1 represents a relatively newer reasoning-oriented LLM, we performed a clinically relevant qualitative review of representative DeepSeek-R1 responses to identify completeness of explanation, citation quality, and clinical appropriateness. The following section presents the responses generated by DeepSeek-R1 to the posed inquiries, followed by the raters' systematic analysis of each answer:

### 3.1 Question 1: what is a patellar dislocation?

#### 3.1.1 Analysis

Patellar dislocation predominantly presents as lateral displacement of the patella. Vertical displacement may occur secondary to quadriceps tendon rupture, whereas intra-articular and congenital patellar dislocations are clinically rare (27, 28). The response comprehensively addresses key clinical aspects, including symptomatology, diagnostic approaches, and therapeutic strategies, thereby providing a foundational understanding for lay readers. However, critical anatomical and functional correlations remain insufficiently elaborated. The physiological lateral patellar glide (normal range: <3/4 patellar width in relaxed knee) is not distinguished from pathological dislocation, which may lead to diagnostic misinterpretation (29). A Tibial Tubercle–Trochlear Groove (TT–TG) distance greater than 20 mm and the prevalence of patella alta (observed in 24%–60% of patients with acute dislocations) are recognized as hallmark indicators of patellofemoral instability (30).

### 3.2 Question 2: under what circumstances does patellar dislocation occur?

#### 3.2.1 Analysis

The response inadequately classifies the etiological factors of patellar dislocation, presenting heterogeneous risk elements in parallel. This unstructured approach leads to conceptual overlaps—for example, anatomical predisposition could be subsumed under non-traumatic causes—resulting in diminished clinical clarity. While the response offers basic anatomical explanations, it lacks discussion of biomechanical mechanisms. Incorporating dynamic injury scenarios (e.g., knee valgus with tibial external rotation during cutting maneuvers, which can precipitate lateral patellar subluxation) would enhance mechanistic understanding for lay inquirers.

Current evidence strongly supports three morphological determinants as risk factors for patellar dislocation: trochlear dysplasia, increased TT–TG distance, and patella alta. In contrast, epidemiological associations remain weakly evidenced. Genetic predisposition requires further validation through large-scale genome-wide association studies (GWAS) (31, 32). Controversial or inconclusive content—such as debated risk factors—should be explicitly acknowledged to prevent the spread of misinformation.

### 3.3 Question 3: what are the clinical manifestations of patellar dislocation?

#### 3.3.1 Analysis

The response accurately delineates the symptomatology associated with patellar dislocation, encompassing both acute manifestations (e.g., hemarthrosis, joint locking) and chronic sequelae secondary to developmental knee deformities (e.g., habitual instability, progressive patellofemoral osteoarthritis). However, a critical omission exists regarding concomitant knee pathologies. Given that patellar dislocation has a lower incidence compared to other sports-related knee injuries [e.g., patellar dislocation 3.3% vs. anterior cruciate ligament (ACL) injury 20.3%] and is frequently associated with multi-ligamentous trauma, the response should incorporate discussion of these additional knee injuries to provide a more comprehensive clinical perspective (30, 33).

### 3.4 Question 4: how is patellar dislocation diagnosed?

#### 3.4.1 Analysis

The response demonstrates logical coherence sufficient to guide the accurate diagnosis of patellar dislocation. However, the physical examination section omits two critical diagnostic maneuvers: the Apprehension Test (to provoke patellar instability) and the Patellar Tilt Test (to assess lateral retinacular tightness) (29). Pain characterization requires a more nuanced interpretation:

- **Persistent pain** may suggest nerve injury (e.g., lumbar radiculopathy) or referred pain.
- **Sharp, intermittent pain** unrelated to specific activities may indicate joint laxity, chondral instability, or impending soft tissue impingement.
- **Activity-induced pain** typically reflects mechanical overload, patellofemoral malalignment, or synovial inflammation (34).

The Sunrise (Merchant) view is employed in axial radiography to accurately assess patellofemoral congruence. Magnetic Resonance Imaging (MRI) provides the highest sensitivity for detecting post-traumatic osteochondral fractures following patellar dislocation. Therefore, routine MRI protocols are recommended for first-time patellar dislocation cases. Recently, a study reported that three-dimensional CT can effectively evaluate the patellofemoral joint, characterize patellar tracking within the trochlear groove, and may aid in guiding the management of patellar dislocation (35). When imaging reveals concomitant pathologies—such as medial patellofemoral ligament (MPFL) avulsion or osteochondral fragments—surgical intervention should be considered, including arthroscopic assessment and MPFL reconstruction as indicated (36).

### 3.5 Question 5: how is patellar dislocation treated?

#### 3.5.1 Analysis

The response appropriately addresses the indications for conservative management and surgical intervention. However, the therapeutic recommendations lack specificity regarding the duration of rest, the optimal timing for initiating strengthening exercises, and the details of the rehabilitation protocol. The postoperative period of 2–4 weeks is a critical phase for rehabilitation, during which range of motion (ROM) exercises should be initiated with the assistance of a brace to limit joint movement. Prolonged immobilization without progressive knee mobilization and muscle strengthening may result in joint stiffness and delayed functional recovery (37). While intra-articular injections—such as hyaluronic acid and platelet-rich plasma (PRP)—have demonstrated efficacy in alleviating symptoms of knee osteoarthritis, their therapeutic value in the management of patellar dislocation remains inconclusive and requires further investigation (38).

### 3.6 Question 6: how effective is conservative treatment of patellar dislocation?

#### 3.6.1 Analysis

The response's assessment of conservative treatment efficacy references outdated review literature (published over 10 years ago), which diminishes the credibility of the evidence cited. However, it is noteworthy that the presented viewpoints align well with current research trends. Significant controversy persists regarding surgical intervention for first-time patellar dislocation, with limited indications for primary surgical management. Existing studies have demonstrated no significant differences in outcomes between surgical and conservative approaches for first-time dislocations without osteochondral injuries or concomitant pathologies, highlighting ongoing clinical equipoise in therapeutic decision-making (39–42). Given the lack of substantial differences in clinical outcomes between these two cohorts, routine surgical intervention is not recommended for acute patellar dislocation in children and adolescents (43).

However, immediate surgical intervention becomes necessary when young patients present with osteochondral detachment secondary to patellar dislocation (44). Surgical stabilization should also be considered for patients who experience recurrent subluxation episodes after six months of conservative management or sustain a second dislocation event (45). Emerging evidence indicates higher rates of redislocation and subluxation associated with conservative approaches compared to surgical stabilization following primary patellar dislocation, suggesting deficiencies in current rehabilitation protocols that require refinement (46–48). Moreover, MPFL reconstruction has demonstrated improved functional outcomes compared to conservative treatment (Kujala score mean difference: 8.10, 95% CI: 6.46–9.75). Although surgical intervention remains the first-line therapeutic strategy for achieving long-term patellofemoral stability in select cases, these findings require validation through multicenter randomized controlled trials with extended follow-up durations (49). Notably, the response also revealed limitations in DeepSeek's information retrieval capabilities, as evidenced by its failure to establish verifiable source linkages to a referenced web page.

### 3.7 Question 7: what are the risks associated with surgical treatment of patellar dislocation?

#### 3.7.1 Analysis

The response comprehensively enumerates general surgical risks (e.g., anesthesia-related complications, infection) as well as procedure-specific risks associated with patellar dislocation surgery, particularly those related to graft failure and postoperative rehabilitation protocols. Meta-analytic evidence indicates a cumulative complication rate of 26.1% following patellar stabilization procedures, underscoring the need for vigilant perioperative risk mitigation strategies. The primary complications of MPFL reconstruction include patellar fracture, postoperative instability, loss of knee flexion, and persistent anterior knee pain. However, current evidence is limited by a paucity of high-level studies evaluating MPFL reconstruction techniques, which restricts the ability to accurately estimate the true incidence of these complications. Furthermore, the lack of quantitative data on complication prevalence underscores the need for additional high-quality investigations to establish robust clinical evidence (50). Given the limitations of AI in accounting for individualized anatomical variations (e.g., TT–TG distance and patellar height indices) and personalized risk profiles (e.g., genetic predispositions and comorbidities), the response's concluding recommendation to consult a physician for surgical risk assessment is appropriately emphasized.

### 3.8 Question 8: what are the surgical methods for patellar dislocation?

#### 3.8.1 Analysis

The evolving understanding of patellar dislocation etiology—such as trochlear dysplasia and lower limb torsional deformities—has driven the development of diverse surgical approaches. The response comprehensively enumerates current surgical options; however, it lacks differentiation between open and arthroscopic techniques. Open MPFL reconstruction demonstrates the highest efficacy in improving Kujala scores and the lowest redislocation rates. Arthroscopic MPFL repair yields the most significant enhancement in Tegner activity scores and substantial reductions in patellar subluxation and redislocation rates, although these improvements are generally less pronounced than those achieved with open procedures. This discrepancy may be attributed to the superior visualization in open surgery, allowing for more precise graft positioning and tension adjustment under direct anatomical exposure (47).

Compared to isolated MPFL reconstruction, the combination of MPFLR and TTO significantly reduces postoperative relative tibial external rotation (rTER), thereby normalizing rTER values. For patients with increased TT–TG distance and abnormal rTER, MPFLR combined with TTO may represent the preferred surgical strategy (51). In adolescents diagnosed with systemic joint hypermobility—such as generalized joint hypermobility (GJH), characterized by ligamentous laxity, hyperextensibility, and hypotonia—MPFLR with TTO can also be considered (52). The combined application of derotational distal femoral osteotomy (D-DFO) and MPFLR has been shown to improve clinical outcomes in patients with recurrent patellar dislocation and excessive femoral anteversion angle (FAA), effectively reducing redislocation rates. In cases involving pathological FAA, adjunctive D-DFO achieves more favorable outcomes in terms of subjective function and patellar stability when compared to isolated MPFLR (53–55). Similarly, in patients with RPD and genu valgum, the combination of MPFLR and distal femoral varus osteotomy (DFVO) demonstrates significant improvements in clinical outcomes—including pain relief and functional scores—along with radiologically validated correction of the valgus deformity (56, 57).

Combined surgical approaches have also shown promising efficacy in managing habitual patellar dislocation. In one study involving 12 adolescent patients, a hybrid protocol incorporating five procedures—lateral soft tissue release, medial soft tissue plication, medial quadriceps advancement, MPFL reconstruction, and rectus femoris insertion reconstruction—resulted in favorable outcomes, with no reported complications such as surgical site infection, joint stiffness, or patellar redislocation (58).

The response appropriately emphasizes the critical role of preoperative imaging assessments (e.g., TT–TG distance measurement, Dejour classification of trochlear dysplasia, and Caton-Deschamps index for patellar height evaluation) in guiding personalized surgical planning. Notably, the inclusion of a disclaimer acknowledging the need for further investigation reflects an understanding of AI's limitations in providing patient-specific recommendations—a nuance that is absent in many other responses. When augmented with “*Web Search*” capabilities, DeepSeek's response cites a recent study, reinforcing its temporal validity and clinical relevance through the integration of cutting-edge evidence (59).

### 3.9 Question 9: how should rehabilitation exercises be conducted post-surgically for patellar dislocation?

#### 3.9.1 Analysis

The response should explicitly address the correlation between surgical techniques and their corresponding rehabilitation protocols. Currently, there is no established consensus regarding optimal postoperative rehabilitation strategies. In patients undergoing TTO, traditional protocols limit weight-bearing for 6–8 weeks to reduce the risk of fixation failure or tibial fracture. However, one study demonstrated that accelerated rehabilitation protocols neither increased complication rates nor adversely affected clinical outcomes in TTO patients with patellar instability (60).

The 2–4-week postoperative window constitutes a critical period for ligament graft remodeling, necessitating the timely initiation of structured rehabilitation. Comparative studies reveal that aggressive mobilization following MPFL reconstruction enhances early functional recovery and reduces quadriceps atrophy in patients with recurrent dislocation, without compromising patellar stability (37).

A structured rehabilitation protocol should include periodic functional assessments of the knee joint throughout the recovery process. Clinicians should be proactively informed as patients approach readiness for high-demand activities (e.g., sport-specific drills, jumping maneuvers) to mitigate reinjury risk. Physician evaluations should incorporate both anatomical integrity verification and psychosocial readiness assessments. Furthermore, regular functional performance testing during rehabilitation can improve patient self-efficacy and help reduce kinesiophobia by providing objective recovery benchmarks (61).

### 3.10 Question 10: how soon can I return to sports after patellar dislocation surgery?

#### 3.10.1 Analysis

This rehabilitation strategy comprehensively addresses the key components of postoperative recovery. Notably, a complete return-to-sport (RTS) testing battery may commence as early as four months postoperatively, with monthly assessments implemented to sustain athlete engagement through visible progress, refine recovery goals, and dynamically adjust rehabilitation programming (62). RTS should be based on individualized criteria rather than assigning a fixed time point. To qualify for RTS, patients must meet the following criteria: (1) absence of pain; (2) absence of effusion; (3) patellofemoral joint stability; (4) full range of motion; (5) nearly symmetric strength (85%–90% of the contralateral limb); and (6) excellent dynamic stability. Ideally, these benchmarks should be met within six weeks of dislocation and by three months after surgery. For participation in high-demand sports (e.g., alpine skiing, soccer, basketball, handball), a limb symmetry index (LSI) ≥90% across functional tests—such as the single-leg squat, Star Excursion Balance Test (SEBT), drop jump test, and side-hop test—is required for medical clearance. Approximately two-thirds of patients return to their pre-injury activity level, with full sport-specific capacity typically regained at an average of 9.7 months post-injury (63, 64).

Overall, the response quality produced by both chatbots was moderate. Compared with ChatGPT-4o, DeepSeek provided a more comprehensive and detailed answer to Q8, offering a wider range of surgical options and adding postoperative management. For Q10, ChatGPT-4o specified return-to-sport requirements based on functional assessment, whereas DeepSeek focused more on a rehabilitation timeline. Notably, neither model mentioned special considerations for skeletally immature patients, who represent a high-risk group for patellar dislocation.

## 4 Discussion

The healthcare landscape in many countries is characterized by underdeveloped medical infrastructure and inadequate per-capita medical resources, which consequently restricts patient access to evidence-based therapeutic interventions (65, 66). In this situation, AI chatbots may function as multidimensional interfaces that concurrently serves as: (1) an evidence-based navigation for patient care pathways; (2) a clinical decision-support adjunct providing supplementary therapeutic annotations; and (3) an interactive continuing medical education platform for healthcare professionals. AI chatbots are convenient tools for health knowledge dissemination. Therefore, this study focused on the readability and professional quality of DeepSeek-R1 and compared its responses with those generated by ChatGPT-4o, with the ultimate goal of using conversational AI to address patient questions and improve disease understanding.

Regarding professional quality, the DISCERN total scores for DeepSeek-R1 and ChatGPT-4o were 49.17 (40.25–59.17) and 50.33 (38.92–58.42), corresponding to a “Fair” level. For these two models, DeepSeek-R1 and ChatGPT-4o obtained scores of DISCERN-S1 [27.33 (26.58–30.75) vs. 27.00 (26.25–30.17)] and DISCERN-S2 [19.00 (8.75–24.08) vs. 19.00 (7.67–22.42)]. A primary contributor appears to be limited source attribution: only DeepSeek-R1 responses to Questions 6 and 8 and the ChatGPT-4o response to Question 10 provided citations, which likely depressed DISCERN Section 1 scores that emphasize clarity, transparency, and accessible evidence. Furthermore, DISCERN Section 2 rewards comprehensive discussion of management options; however, our single-focus prompts (each targeting a specific aspect of patellar dislocation) naturally elicited narrowly scoped answers rather than an exhaustive appraisal of alternatives, risks, and shared decision-making considerations, thereby reducing some Section 2 subsection scores. Notably, when citations were present, citation relevance remained a concern; for example, in Question 6, a low-relevance reference did not substantiate the associated claims, highlighting that AI-generated references require independent validation before clinical use, citation presence alone is insufficient without verifiability and topical alignment, citation generation by LLMs should not be considered equivalent to evidence verification. No statistically significant difference between two models was detected for DISCERN total score (*P* = 0.767), DISCERN-S1 (*P* = 0.165) or DISCERN-S2 (*P* = 1.00). A non-significant *p*-value should not be interpreted as evidence of equivalence. With only 10 paired items and highly clustered scores, the study is underpowered to detect small between-model differences.

For readability, DeepSeek-R1 produced significantly higher textual complexity and poorer readability than ChatGPT-4o (*P* < 0.05). DeepSeek-R1's “DeepThink” reasoning mode—which employs chain-of-thought prompting to generate more detailed, step-by-step clinical reasoning—may inherently produce longer sentences and more technical terminology. The detail of the mechanisms underlying this difference remain uncertain and may involve model architecture, training strategy, reasoning processes, or decoding parameters. Both models showed significantly poorer readability than the NIH-recommended sixth-grade reading level (*P* < 0.05; Table 1). It is particularly problematic because patellar dislocation disproportionately affects children and adolescents, for whom patient-facing information should be adapted to appropriate health-literacy levels. Some anatomical phrasing such as “tight iliotibial band (IT band) or lateral retinaculum pulling the patella outward” exceeds patients' biomedical literacy. Prefacing user queries with phrases like “As a middle school student, I need to understand…” may prompt lexical adaptation and improve response readability. Future studies should verify the influence of prompt words on the responses of AI chatbots. Also, the use of simplified language, avoidance of unnecessary medical jargon, and explanations tailored to specific literacy levels may improve accessibility while preserving clinical accuracy.

Many prior studies support our results that AI chatbots can generate clinically relevant answers of acceptable information quality, yet their outputs often remain too complex for the general population and their DISCERN performance may be constrained by limited citation transparency (9, 14, 15, 22, 67). At the same time, some orthopaedic investigations report more favorable performance, suggesting that in certain contexts AI-generated answers (e.g., evaluations in total hip arthroplasty and total ankle arthroplasty) can be clinically acceptable and sufficiently complete for common patient concerns (10, 11, 16). In contrast to the low readability observed in our study, a subset of evidence points to potential advantages of AI over traditional medical sources under specific conditions. In oncology, chatbot responses to cancer questions from social media have been rated higher in quality, empathy, and readability than physician responses (5). Moreover, AI has been shown to improve the readability of existing medical materials when used explicitly for rewriting or simplification, demonstrating a practical pathway to reduce literacy burden without necessarily compromising information integrity (4, 7). Taken together, our patellar-dislocation results align with the broader literature in showing persistent deficits in verifiable sourcing and reading-level control, while adding evidence that between-model readability differences can be domain- and prompt-sensitive rather than stable across clinical topics.

A key limitation of this study is the single-turn, single-generation evaluation design. Although this approach enabled standardized comparison between models as other similar surveys, it does not fully represent real-world interactions, where patients may engage in iterative conversations, request clarification, or modify their questions based on previous responses. Such multi-turn interactions may allow LLMs to correct misunderstandings, provide additional explanations, or introduce new sources of variability. Therefore, our findings should be interpreted as an evaluation of initial AI-generated responses rather than the complete conversational capability of these systems. Another key limitation is the small sample size. Given that this study analyzes each AI's response and its characteristics, we prioritized depth of assessment over a larger number of answers. The study included only 10 responses, which limits statistical power and the ability to detect small between-model differences. Absence of statistical significance should not be interpreted as equivalence. Given the small number of paired observations, clinically meaningful differences may remain undetected. The small item set may also reduce generalizability, as the selected questions cannot fully represent the breadth of real-world clinical scenarios in patellar dislocation. Another limitation is that the evaluated LLMs ignore adolescents' development characteristics. Given the high incidence of patellar dislocation in adolescents, treatment decisions in this population require consideration of growth disturbance risks, age-specific treatment and surgical decision-making. Failure to address these factors may lead to incomplete or potentially misleading patient education. Additionally, the exploratory qualitative analysis focused only on DeepSeek-R1. Therefore, these findings should be interpreted as exploratory observations rather than direct comparative evidence. Future work should incorporate multi-turn, multi-generation frameworks, more samples with repeated sampling, longitudinal assessment, enforced citation standards and expanded evaluation to better quantify both clinical utility and reliability.

## 5 Conclusion

In this paired, single-turn evaluation of AI-generated clinical guidance for patellar dislocation, both DeepSeek-R1 and ChatGPT-4o achieved “Fair” overall information quality as assessed by DISCERN. No statistically significant between-model differences were detected for DISCERN total score, DISCERN-S1, or DISCERN-S2, suggesting no significant statistical difference between models within our limited sample study. For readability, both models produced text far above recommended patient-reading levels, and DeepSeek-R1 consistently generated more complex, less readable responses than ChatGPT-4o. In addition, verifiable citations were uncommon, and the single-turn/single-generation design likely constrained coverage of management alternatives. These findings suggest that, while both systems can produce relevant responses of moderate quality, their outputs should be regarded as adjunctive information rather than stand-alone clinical guidance, particularly given the high incidence of patellar instability and development characteristics of children and adolescents. Future work should incorporate multi-turn, multi-generation frameworks, more samples with repeated sampling, longitudinal assessment, enforced citation standards and expanded evaluation to better quantify both clinical utility and reliability.

## Data Availability

The original contributions presented in the study are included in the article/Supplementary Material, further inquiries can be directed to the corresponding author.
