# Supplementary material for: Evaluating artificial intelligence-generated clinical guidance for patellar dislocation: accuracy, readability and qualitative appraisal of DeepSeek-R1 responses
Source: Front Digit Health. 2026 Sep 29;8:1719101. doi: 10.3389/fdgth.2026.1719101 (PMC13658195; doi:10.3389/fdgth.2026.1719101)
Supplement: Supplementary file 1 [file Supplementaryfile1.docx]

Supplementary Material 1

**Question:1.** **What is a patellar dislocation?**

**ChatGPT-4o response:**

A **patellar dislocation** occurs when the **kneecap (patella)** moves out of its normal position in the **groove of the femur (thighbone)**, typically sliding **laterally (to the outside)** of the knee.

**🔍 Anatomy Overview**

- The **patella** sits in the **trochlear groove** of the femur.
- It is stabilized by:
  - **Ligaments** (especially the medial patellofemoral ligament, or MPFL)
  - **Muscles**, especially the **quadriceps**
  - The shape of the **bony groove** itself

**⚠️ What Happens in a Dislocation?**

- The patella is forced out of its groove, usually due to:
  - A **traumatic injury** (e.g., twisting the knee while the foot is planted)
  - A **direct blow** to the knee
  - An **abnormality** in knee anatomy (like a shallow trochlear groove or ligament laxity)

**🧬 Risk Factors**

- Young age (teens and young adults)
- Female sex (due to wider pelvis and anatomical differences)
- Prior patellar dislocation or instability
- Sports involving pivoting, jumping, or quick direction changes (e.g., soccer, basketball)
- Structural abnormalities:
  - Shallow femoral groove
  - High-riding patella (patella alta)
  - Ligamentous laxity (e.g., Ehlers-Danlos syndrome)

**🩺 Symptoms**

- Sudden **pain** and **swelling**
- **Visible deformity** of the kneecap (often to the outer side)
- **Inability to straighten** the knee
- Sensation of the knee “giving way”

**🏥 Diagnosis**

- Clinical evaluation
- Imaging:
  - **X-rays** to confirm dislocation and rule out fractures
  - **MRI** to assess ligament/tendon damage, especially to the MPFL

**🛠️ Treatment**

**Initial dislocation (without complications):**

- **Closed reduction** (manual repositioning)
- **Bracing** and physical therapy
- Gradual return to activity

**Recurrent dislocations or ligament injury:**

- **Surgical intervention**, such as:
  - MPFL reconstruction
  - Trochleoplasty (reshaping the femoral groove)
  - Tibial tubercle realignment

**🏃 Prognosis**

- Many recover well with **non-operative** treatment after a first-time dislocation.
- However, recurrence rates can be high, especially in younger athletes.
- Repeated dislocations may lead to **cartilage damage** and **arthritis** over time.

Let me know if you’d like a visual aid or a summary chart for clinical use.

**Question:2.** **Under what circumstances does patellar dislocation occur?**

**ChatGPT-4o response:**

Patellar dislocation occurs when the patella (kneecap) moves out of its normal position, typically slipping laterally (to the outside of the knee). This usually happens due to trauma or instability and can occur under several circumstances:

**1. Acute Trauma (Most Common in First-Time Dislocation)**

- **Direct blow to the knee**, especially when the knee is slightly flexed and the foot is planted (e.g., during a football tackle).
- **Sudden twisting movements**, such as pivoting or turning quickly while the foot is fixed, often seen in sports like basketball, soccer, or gymnastics.

**2. Anatomical and Biomechanical Predispositions**

Some individuals are more prone to patellar dislocation due to structural factors, such as:

- **Shallow trochlear groove** (where the patella sits in the femur).
- **Patella alta** (high-riding patella).
- **Increased Q angle** (angle between the hip and knee).
- **Ligament laxity** or **hypermobile joints** (common in connective tissue disorders like Ehlers-Danlos syndrome).
- **History of previous dislocation**, which increases recurrence risk.

**3. Congenital or Developmental Conditions**

- In some cases, children may have **congenital patellar instability**, leading to dislocations during normal activities or with minimal trauma.

**4. Recurrent Dislocation (Chronic Instability)**

- After an initial dislocation, if the **medial patellofemoral ligament (MPFL)** or surrounding stabilizers do not heal properly, patients may experience **recurrent dislocations** with minor movements or even normal walking.

**5. During Sports or Physical Activities**

- Activities involving **jumping**, **cutting**, or **lateral movements**.
- Sports with rapid direction changes like **basketball**, **volleyball**, or **skiing**.

If you'd like, I can also provide an overview of risk factors, diagnosis, or treatment options.

**Question 3.** **What are the clinical manifestations of patellar dislocation?**

**ChatGPT-4o response:**

The **clinical manifestations of patellar dislocation** typically occur suddenly and are often easy to recognize due to the nature of the injury. Here are the key signs and symptoms:

**1. Sudden Onset of Knee Pain**

- Patients usually experience a **sharp, intense pain** at the front of the knee, especially around or just below the patella (kneecap).
- The pain is often severe enough to limit weight-bearing or walking.

**2. Visible Deformity**

- In **lateral dislocations** (most common), the patella visibly shifts or rests on the outside (lateral side) of the knee.
- In some cases, it may **spontaneously relocate**, but the knee may still appear swollen or abnormal afterward.

**3. Swelling and Effusion**

- Rapid **swelling (hemarthrosis)** occurs within hours due to bleeding into the joint space.
- The knee may feel tight and difficult to move due to fluid accumulation.

**4. Loss of Function**

- Difficulty or inability to **straighten or bend the knee**.
- Patients often report feeling like the knee will “give out.”

**5. Tenderness and Bruising**

- The medial (inner) side of the knee may be **tender to palpation**, especially at the **medial patellofemoral ligament (MPFL)** attachment.
- **Bruising** around the patella and medial thigh may appear within hours to days.

**6. Sensation of Giving Way or Instability**

- After the dislocation relocates (either spontaneously or manually), patients may describe a **persistent feeling of instability** or “giving way,” especially during turning or pivoting motions.

**7. Audible "Pop" or Shift**

- Many patients report hearing or feeling a **“pop”** at the time of injury, which is often the moment the patella dislocates.

**8. Limited Range of Motion**

- Both **active and passive range of motion** are limited due to pain and joint effusion.

**Additional Considerations**

- **First-time dislocations** are usually trauma-related (e.g., twisting injuries during sports).
- **Recurrent dislocations** may have milder symptoms but are often associated with underlying anatomical predispositions (e.g., patella alta, trochlear dysplasia).

Let me know if you’d like a comparison with subluxation or a breakdown by mechanism (traumatic vs. atraumatic).

**Question 4. How is patellar dislocation diagnosed?**

**ChatGPT-4o response:**

Patellar dislocation is typically diagnosed based on a combination of the patient’s history, physical examination findings, and imaging studies. Here's how the diagnostic process usually works:

**1. Clinical History**

Physicians begin by asking about:

- **Mechanism of injury**: Often a twisting injury with the foot planted and knee in slight flexion.
- **Symptoms at the time of injury**: Sudden pain, knee giving way, or a feeling of the kneecap "popping out".
- **Previous episodes**: A history of recurrent dislocations or instability.
- **Associated findings**: Swelling, difficulty bearing weight, or locking.

**2. Physical Examination**

Key physical signs include:

- **Visible deformity**: The patella may be obviously displaced (typically laterally) during acute dislocation.
- **Tenderness**: Especially over the medial patellofemoral ligament or medial patella.
- **Swelling or joint effusion**: Common in acute cases.
- **Positive apprehension test**: When the examiner gently pushes the patella laterally, the patient shows fear or resistance, indicating instability.
- **Gait and range of motion**: Limited or painful motion, limping gait.

**3. Imaging Studies**

Imaging confirms the diagnosis and evaluates for associated injuries:

- **X-rays**:
  - *Anteroposterior, lateral, and sunrise (axial) views* are standard.
  - Look for lateral displacement of the patella or osteochondral fragments.
- **MRI**:
  - Evaluates **soft tissue damage**, including:
    - Medial patellofemoral ligament (MPFL) injury
    - Bone bruising on the lateral femoral condyle and medial patella
    - Osteochondral lesions
  - Helpful for surgical planning and assessing recurrent dislocations.
- **CT scan** (less commonly used):
  - Provides detailed bone alignment assessment and measures anatomical predispositions (e.g., trochlear dysplasia, patella alta, or increased tibial tubercle–trochlear groove (TT-TG) distance).

**Summary**

Patellar dislocation is diagnosed primarily through a combination of patient history, physical examination (e.g., apprehension test), and imaging — especially X-rays and MRI. These tools help confirm the dislocation, assess the extent of soft tissue damage, and guide treatment planning.

Let me know if you'd like diagnostic criteria for *recurrent* dislocation or *treatment options* as well.

**Question 5. How is patellar dislocation treated?**

**ChatGPT-4o response:**
Treatment for **patellar dislocation** depends on whether it's a **first-time dislocation** or a **recurrent** one, and whether there is **associated injury** (such as cartilage damage or ligament tearing). Here's an overview:

**🏥 Initial Management (First-Time Dislocation)**

**1. Immediate Care (Acute Phase)**

- **Reduction**: The patella is usually manually pushed back into place by a healthcare professional (closed reduction).
- **RICE Protocol**:
  - **Rest**
  - **Ice**
  - **Compression**
  - **Elevation**
- **Immobilization**:
  - A **knee brace or splint** may be used for 2–3 weeks to limit movement.
- **Crutches**: To avoid weight bearing if there's significant pain or instability.

**2. Imaging**

- **X-ray** to assess bony anatomy.
- **MRI** to check for:
  - Medial patellofemoral ligament (MPFL) tear
  - Osteochondral injuries
  - Loose fragments

**3. Physical Therapy**

- Focuses on:
  - **Quadriceps strengthening** (especially vastus medialis obliquus)
  - **Hip and core muscle strengthening**
  - **Balance and proprioception training**

**🔁 Management of Recurrent Dislocation or Severe Injury**

**1. Surgical Intervention**

May be considered if:

- There are **loose bone or cartilage fragments**
- MPFL is severely torn or stretched
- There is **underlying anatomic predisposition** (e.g., trochlear dysplasia, patella alta, or malalignment)

**Surgical options include**:

- **MPFL reconstruction**: Most common; restores medial patellar stability.
- **Tibial tubercle transfer (Fulkerson osteotomy)**: Realigns the extensor mechanism.
- **Trochleoplasty**: Deepens the femoral groove in cases of dysplasia.
- **Lateral release**: Rarely used alone now but may be part of combined procedures.

**🦵 Rehabilitation Post-Treatment**

- Return to sport or strenuous activity typically takes **3–6 months**, depending on severity and whether surgery was needed.
- Emphasis is on:
  - Full **range of motion**
  - Restored **strength and stability**
  - **Pain-free function**

**🧠 Summary**

| **Type of Dislocation** | **Treatment Options** |
| --- | --- |
| **First-time, mild** | Non-operative: RICE, bracing, PT |
| **Recurrent or severe** | May need surgery + structured rehab |
| **With osteochondral damage** | Surgery often indicated |

Let me know if you'd like a treatment comparison table or rehabilitation protocol summary.

**Question 6. How effective is conservative treatment of patellar dislocation?**

**ChatGPT-4o response:**

Conservative (non-surgical) treatment for **patellar dislocation**, especially a **first-time lateral patellar dislocation**, is often effective in many patients—particularly those without significant anatomical abnormalities or intra-articular injuries. However, its success depends on several patient- and injury-specific factors.

**💡 General Effectiveness of Conservative Treatment**

Conservative treatment usually includes:

- **Closed reduction** (if the patella remains dislocated),
- **Immobilization** (brief period of knee bracing or taping),
- **Physical therapy** focusing on:
  - Quadriceps strengthening (especially vastus medialis obliquus),
  - Hip and core strengthening,
  - Patellar stabilization and proprioceptive training.

**Outcomes**:

- Approximately **60–70%** of first-time dislocations treated conservatively **do not redislocate**.
- Many patients **regain full function** and return to sports/activities.
- However, the **redislocation rate** can reach **15–45%**, particularly in young, active individuals or those with risk factors (e.g., trochlear dysplasia, patella alta, ligamentous laxity).

**🔄 When Conservative Treatment May Fail**

Risk factors for **recurrence after conservative treatment**:

- Age < 20 years at first dislocation,
- Anatomical predispositions (e.g., shallow trochlear groove),
- Previous contralateral patellar instability,
- High activity level (e.g., athletes in pivoting sports).

In these patients, **surgical stabilization** (e.g., MPFL reconstruction) may be considered earlier.

**📝 Evidence from Research**

1. **Cochrane Review (2011)**:
   - No strong evidence that surgery is superior for first-time dislocation.
   - Conservative treatment associated with lower complication rates.
2. **Randomized trials**:
   - Some show **lower recurrence rates with early surgery**, but **no difference in functional outcomes or patient satisfaction** compared to nonoperative care.
3. **Recent guidelines**:
   - Recommend starting with nonoperative treatment in most first-time cases unless there is:
     - Osteochondral fragment,
     - Severe anatomic risk factors,
     - Locked dislocation.

**✅ Summary**

| **Aspect** | **Conservative Treatment** |
| --- | --- |
| Effectiveness | Good in 60–70% of cases |
| Redislocation Risk | Moderate (15–45%) |
| Best Candidates | First-time dislocation without major anatomic risk |
| Treatment Components | Rest, brace, physiotherapy |
| Surgery Consideration | For high-risk or recurrent cases |

If you have a specific case or patient profile in mind (e.g. age, recurrence, MRI findings), I can tailor the recommendations more precisely.

**Question 7. What are the risks associated with surgical treatment of patellar dislocation?**

**ChatGPT-4o response:**

Surgical treatment for patellar dislocation—typically involving procedures such as medial patellofemoral ligament (MPFL) reconstruction, tibial tubercle transfer, or trochleoplasty—can be effective, especially in cases of recurrent dislocations. However, like all surgeries, it carries certain risks. These include:

**🩸 Surgical and Immediate Postoperative Risks**

- **Infection**: A risk with any surgical procedure, though relatively low with proper sterile technique and antibiotics.
- **Bleeding or hematoma formation**: May require drainage if significant.
- **Deep vein thrombosis (DVT)**: Risk increases with prolonged immobility post-surgery.

**🦵 Joint- and Function-Specific Complications**

- **Stiffness or decreased range of motion**: May result from over-tightening structures or inadequate rehabilitation.
- **Persistent instability or recurrent dislocation**: Surgery may not fully resolve the problem, especially in cases with complex underlying anatomy.
- **Maltracking or overcorrection**: Misalignment of the patella can lead to new problems, such as medial instability or increased joint wear.
- **Arthrofibrosis**: Excessive scar tissue formation limiting movement.

**🦴 Hardware or Structural Issues**

- **Hardware irritation**: Screws or anchors used in bone fixation may cause pain or irritation, occasionally requiring removal.
- **Fracture**: Rarely, bone cuts (e.g., tibial tubercle osteotomy) can lead to fractures during or after surgery.
- **Graft failure**: In MPFL reconstruction, the graft can fail or stretch over time, especially if rehab is too aggressive too early.

**⏳ Long-Term Complications**

- **Patellofemoral osteoarthritis**: Surgical changes in patellar mechanics may increase the risk of degenerative joint changes over time.
- **Chronic pain**: Even with correct surgical technique, some patients experience long-term anterior knee pain.

**🧠 Psychological and Rehabilitation Challenges**

- **Fear of re-injury**: Can delay return to sport or reduce performance.
- **Non-compliance with rehabilitation**: Increases risk of complications or suboptimal outcomes.

**⚖️ Risk-Benefit Balance**

Surgery is typically reserved for:

- Recurrent patellar dislocations
- Significant anatomical abnormalities (e.g., trochlear dysplasia)
- Failure of conservative treatment

When indicated and performed correctly, surgical treatment often improves outcomes—but patient selection, surgical technique, and postoperative rehab are critical to success.

Would you like a comparison table between surgical and conservative outcomes or specific data on complication rates?

**Question 8. What are the surgical methods for patellar dislocation?**

**ChatGPT-4o response:**

Surgical treatment of **patellar dislocation** is generally considered in cases of recurrent dislocations, failure of conservative management, or in acute dislocation with associated structural damage (e.g., osteochondral fracture, MPFL tear). Below are the main **surgical methods** used to treat patellar instability:

**1. Medial Patellofemoral Ligament (MPFL) Reconstruction**

- **Most common surgery** for recurrent lateral patellar dislocation.
- Reconstructs the torn or lax MPFL, the primary soft tissue restraint to lateral patellar displacement.
- Often performed using autograft (e.g., hamstring tendon).
- Can be done arthroscopically or via mini-open approach.
- May be combined with bony procedures if needed.

**2. Tibial Tubercle Osteotomy (TTO)**

- Indicated when there is **abnormal patellar tracking**, excessive lateralization of the tibial tubercle (high TT-TG distance), or **patella alta**.
- The tibial tubercle is cut and repositioned medially and/or distally.
- Common techniques include:
  - **Elmslie-Trillat procedure** (medialization)
  - **Fulkerson osteotomy** (anteromedialization)

**3. Trochleoplasty**

- Reshapes a shallow or dysplastic trochlear groove.
- Indicated in patients with **high-grade trochlear dysplasia**.
- More technically demanding and typically reserved for severe instability unresponsive to other treatments.

**4. Lateral Release (Less common now)**

- Involves cutting tight lateral retinacular structures to reduce lateral pull on the patella.
- Rarely used as an isolated procedure now; often reserved for patients with excessive lateral tightness **in combination** with other procedures.
- Can lead to complications like medial instability if overdone.

**5. Osteochondral Fragment Fixation or Removal**

- Performed in acute dislocation with **osteochondral fractures**.
- Loose fragments are either removed or fixed back, depending on size, location, and viability.

**6. Combined Procedures**

- Many patients benefit from a **combination** of the above procedures tailored to individual anatomic risk factors (e.g., MPFL reconstruction + TTO).

**Surgical Approach Considerations:**

- The choice of procedure depends on:
  - Frequency of dislocation
  - Anatomical abnormalities (e.g., trochlear dysplasia, TT-TG distance, patella alta)
  - Patient age and activity level
  - Imaging findings (MRI, CT)
  - Prior treatments

If you'd like, I can also help with visuals or decision algorithms often used to guide surgical choices.

**Question 9. How should rehabilitation exercises be conducted post-surgically for patellar dislocation?**

**ChatGPT-4o response:**

Rehabilitation following surgical treatment for patellar dislocation is essential for restoring stability, function, and preventing recurrence. A well-structured rehab program progresses through several **phases**, each with specific goals and exercise strategies. Here’s a typical outline:

**Phase 1: Immediate Postoperative Phase (Weeks 0–2)**

**Goals:**

- Protect surgical repair
- Minimize swelling and pain
- Restore quadriceps activation
- Maintain patellar mobility

**Typical Interventions:**

- **Immobilization:** Knee brace locked in extension for ambulation
- **Weightbearing:** Partial to full as tolerated (per surgeon's protocol)
- **Exercises:**
  - Ankle pumps
  - Quadriceps sets
  - Straight leg raises
  - Patellar mobilizations (gentle medial-lateral)
  - Passive/active-assisted ROM (usually 0–90° flexion allowed by end of week 2)

**Phase 2: Early Range of Motion and Strengthening (Weeks 2–6)**

**Goals:**

- Improve ROM gradually
- Begin gentle strengthening
- Normalize gait

**Typical Interventions:**

- **Progressive ROM:** Aim for 120° flexion by week 6
- **Gait training:** Transition from crutches to full weightbearing with normalized gait
- **Strengthening:**
  - Closed kinetic chain exercises (e.g., mini squats, heel raises)
  - Hip abductor and core strengthening
  - Stationary cycling (low resistance)

**Phase 3: Intermediate Strengthening and Proprioception (Weeks 6–12)**

**Goals:**

- Full ROM
- Restore muscular strength
- Improve neuromuscular control

**Typical Interventions:**

- **Exercises:**
  - Leg press (partial range initially)
  - Step-ups/step-downs
  - Balance board training, single-leg stance
  - Treadmill walking

**Phase 4: Advanced Strengthening and Functional Training (Weeks 12–20)**

**Goals:**

- Return to full functional strength
- Prepare for return to sports

**Typical Interventions:**

- **Plyometric drills** (jump squats, box jumps)
- **Agility training** (ladder drills, cutting maneuvers)
- **Sport-specific exercises**
- **Running program** (if appropriate)

**Phase 5: Return to Sport (After 4–6 Months)**

**Criteria:**

- Full ROM
- ≥90% strength symmetry
- No pain, instability, or swelling
- Cleared by surgeon and physical therapist

**Examples:**

- Gradual reintroduction to practice
- Continue neuromuscular training
- Preventive strengthening for quads and hip stabilizers

**Important Considerations**

- **Individualization:** Adjust for type of surgery (e.g., MPFL reconstruction vs. trochleoplasty)
- **Patient compliance:** Critical for successful outcome
- **Surgeon protocol:** Always follow specific surgical protocol variations

Let me know if you'd like a printable rehab protocol or need it tailored to a specific surgery.

**Question 10. How soon can I return to sports after patellar dislocation surgery?**

**ChatGPT-4o response:**

How soon you may return to sports after surgery for a dislocated kneecap (patellar dislocation) depends a lot on the severity, the type of surgery, how well your knee heals, and how carefully you follow rehab — but current research gives a rough guideline you can use to discuss with your surgeon / physiotherapist. Here’s a breakdown based on what’s known.

**✅ What determines “when you’re ready”**

Most experts agree that you should *not* return to sport simply based on how much time has passed since surgery — instead, you should meet **functional and clinical criteria**. Typical requirements before returning to sport include:

- No pain or swelling/effusion in the knee ([PMC](https://pmc.ncbi.nlm.nih.gov/articles/PMC4169614/?utm_source=chatgpt.com))
- Full, pain-free range of motion in the knee joint ([PMC](https://pmc.ncbi.nlm.nih.gov/articles/PMC4169614/?utm_source=chatgpt.com))
- Strength of the operated leg — especially quadriceps and other stabilizers — close to that of the other leg (often ≥ 85‑90 %) ([PMC](https://pmc.ncbi.nlm.nih.gov/articles/PMC4169614/?utm_source=chatgpt.com))
- Good dynamic stability: proper balance, control, ability to absorb force — important especially for sports with jumping, cutting, or rapid direction changes. ([PMC](https://pmc.ncbi.nlm.nih.gov/articles/PMC4169614/?utm_source=chatgpt.com))

A “time‑only” rule is insufficient. ([PMC](https://pmc.ncbi.nlm.nih.gov/articles/PMC9789273/?utm_source=chatgpt.com))

**📅 Typical Recovery / Sport‑Return Timelines After Surgery**

Based on recent studies and reviews, general timelines look like this:

- According to a 2022 review, **comprehensive return‑to‑sport testing can begin as early as about 4 months after surgery**. ([PMC](https://pmc.ncbi.nlm.nih.gov/articles/PMC9789273/?utm_source=chatgpt.com))
- Many patients return to sport around **6–7 months post‑operatively**. In one collection of 130 patients, ~ 92% returned to sport at ~7 months, though only ~78% regained their pre-injury level. ([orthopedicreviews.openmedicalpublishing.org](https://orthopedicreviews.openmedicalpublishing.org/article/146313-research-progress-on-return-to-sport-after-surgery-for-lateral-patellar-dislocation?utm_source=chatgpt.com))
- Other sources recommend a **range of 6 to 12 months** for full return to sports, especially for sports requiring high knee demands (jumping, cutting, pivoting). ([search.mskdoctors.com](https://search.mskdoctors.com/doctors/charlotte-barker/articles/bouncing-back-athletes-road-to-return-after-a-patellar-dislocation?utm_source=chatgpt.com))
- Some rehabilitation‑protocol–based sources indicate that **full knee range of motion and weight‑bearing are often restored by 6 weeks**, but this only marks early functional recovery, not sport readiness. ([ijspt.scholasticahq.com](https://ijspt.scholasticahq.com/article/141128?utm_source=chatgpt.com))

**⚠️ Why there’s variability — and what to watch out for**

- Recovery differs depending on the exact surgical procedure (e.g. soft‑tissue reconstruction vs. other repairs), whether there was cartilage damage, bone injury, or repeated dislocations. ([orthopedicreviews.openmedicalpublishing.org](https://orthopedicreviews.openmedicalpublishing.org/article/146313-research-progress-on-return-to-sport-after-surgery-for-lateral-patellar-dislocation?utm_source=chatgpt.com))
- Even with surgery, only a fraction of patients (in some studies roughly two‑thirds) return to the same sport level as before. ([PMC](https://pmc.ncbi.nlm.nih.gov/articles/PMC4169614/?utm_source=chatgpt.com))
- Returning too early — before strength, stability, and functional control are restored — increases risk of re-injury or further instability. ([www.xphysio.com.au](https://xphysio.com.au/physiotherapys-role-in-healing-kneecap-dislocation/?utm_source=chatgpt.com))

**🎯 What this means for you — a practical guideline**

If you’ve had surgery for a patellar dislocation, a **realistic goal for returning to sport** would likely be **around 6 to 9 months after surgery**, assuming you meet the objective criteria (full motion, strength symmetry, no pain/swelling, knee stability, good neuromuscular control).

You might begin **sport‑specific training / testing around 4 months**, but that doesn’t necessarily mean full competitive play.

The exact timing depends heavily on:

- how well your knee is healing,
- adherence to the rehabilitation / strengthening program,
- what kind of sport you’re aiming to return to (low‑impact vs. cutting/jumping),
- and guidance from your surgeon / physical therapist.

If you like — I can pull up **recent studies (past 5 years)** on return‑to‑sports after patellar dislocation surgery and show you **statistics by sport type** (e.g. soccer, basketball, running).
That might give you a better idea of what to expect depending on what you play.
