# Supplementary material for: Evaluating artificial intelligence-generated clinical guidance for patellar dislocation: accuracy, readability and qualitative appraisal of DeepSeek-R1 responses
Source: Front Digit Health. 2026 Sep 29;8:1719101. doi: 10.3389/fdgth.2026.1719101 (PMC13658195; doi:10.3389/fdgth.2026.1719101)
Supplement: Supplementary file 2 [file Supplementaryfile2.docx]

Supplementary Material 2

**Question:1.** **What is a patellar dislocation?**

**DeepSeek-R1 response:**

**Patellar Dislocation: An Overview**

**Definition:**
A patellar dislocation occurs when the kneecap (patella) is forcibly displaced from its normal position within the femoral groove (trochlea) of the thigh bone (femur). This typically results in the patella shifting laterally (to the outside of the knee), disrupting knee function.

**Anatomy Involved:**
The patella, embedded in the quadriceps tendon, glides within the femoral groove during knee movement. Stability is provided by ligaments (e.g., medial patellofemoral ligament, MPFL), tendons, and muscle support. Dislocation often damages these structures, particularly the MPFL.

**Causes:**

- **Trauma:** Direct impact (e.g., sports collision) or sudden twisting (e.g., changing direction).
- **Anatomical Factors:** Shallow femoral groove, patellar alta (high-riding patella), genu valgum (knock knees).
- **Muscle Imbalance/Weakness:** Weak quadriceps (especially vastus medialis) or tight lateral structures.
- **Ligament Laxity:** Seen in conditions like Ehlers-Danlos syndrome.
- **Risk Groups:** Adolescents, females, athletes (e.g., basketball, soccer).

**Symptoms:**

- Immediate, severe pain and swelling.
- Visible deformity ("empty" femoral groove) or lateral displacement.
- Inability to straighten the knee or bear weight.
- Popping sensation during injury; instability post-reduction.

**Diagnosis:**

- **Physical Exam:** Assessment of alignment, tenderness, and ligament integrity.
- **Imaging:**
  - **X-rays:** Identify displacement, fractures, or bony anomalies.
  - **MRI:** Evaluate soft tissue damage (MPFL, cartilage).

**Treatment:**

1. **Reduction:** Manual realignment by a healthcare professional, often with gentle knee extension.
2. **Immobilization:** Brace or splint for 2–3 weeks to allow healing.
3. **Rehabilitation:** Physical therapy focusing on quadriceps (VMO) strengthening and flexibility.
4. **Surgery:** Indicated for recurrent dislocations, osteochondral fractures, or MPFL repair/reconstruction.

**Complications:**

- **Recurrent Dislocations:** Higher risk after initial injury.
- **Cartilage Damage:** Leading to patellofemoral arthritis.
- **Chronic Instability/Pain:** Affecting mobility and quality of life.

**Prevention:**

- Strengthening exercises (quadriceps, hamstrings).
- Proprioceptive training and sport-specific techniques.
- Bracing/taping for high-risk activities.
- Addressing anatomical issues surgically if recurrent.

**Distinction from Similar Injuries:**

- **Subluxation:** Partial displacement with spontaneous reduction.
- **Knee Dislocation:** Rare, severe injury involving tibiofemoral joint disruption.

This comprehensive approach ensures understanding of patellar dislocation, emphasizing both acute management and long-term strategies to mitigate recurrence and complications.

**Question:2.** **Under what circumstances does patellar dislocation occur?**

**DeepSeek-R1 response:**

Patellar dislocation occurs when the kneecap (patella) is displaced from its normal position in the trochlear groove of the femur, typically toward the lateral side. The circumstances under which this happens can be categorized as follows:

**1. Traumatic Injury**

- **Sudden Impact or Twist**: Direct blows to the knee (e.g., in contact sports) or abrupt twisting motions (e.g., pivoting in soccer, basketball, or skiing).
- **Awkward Landing**: Jumping from a height or landing with the knee in a valgus position (knock-kneed).

**2. Anatomical Predisposition**

- **Shallow Trochlear Groove**: A poorly developed femoral groove reduces patellar stability.
- **Patellar Dysplasia**: Abnormal patellar shape.
- **Patella Alta**: A high-riding patella that sits above the trochlear groove.
- **Increased Q-Angle**: Wider hips (common in females) create a larger angle between the femur and tibia, pulling the patella laterally.
- **Ligamentous Laxity**: Genetic or acquired looseness of ligaments (e.g., Ehlers-Danlos syndrome).

**3. Muscle Imbalance or Weakness**

- **Weak Vastus Medialis Obliquus (VMO)**: Underdeveloped inner quadriceps muscle fails to counteract lateral forces.
- **Tight Lateral Structures**: Tight iliotibial band (IT band) or lateral retinaculum pulling the patella outward.

**4. Previous Dislocation or Instability**

- Recurrent dislocations weaken medial stabilizers (e.g., medial patellofemoral ligament/MPFL), increasing risk of future episodes.

**5. Congenital/Developmental Factors**

- **Developmental Dysplasia of the Hip (DDH)**: Altered lower limb alignment.
- **Flat Feet (Pes Planus)**: Alters leg biomechanics, increasing lateral strain on the patella.

**6. Activity-Related Risks**

- High-risk sports involving jumping, cutting, or rapid direction changes (e.g., gymnastics, volleyball, rugby).

**7. Non-Traumatic Causes**

- Minor movements (e.g., standing up) in individuals with severe underlying instability or connective tissue disorders.

**Symptoms During Dislocation**

- Sudden pain, audible "pop," visible deformity, swelling, and inability to straighten the knee.

**Key Takeaway**

Patellar dislocation often results from a combination of acute trauma and chronic risk factors (anatomical, muscular, or genetic). Adolescents, females, and athletes are particularly susceptible. Recurrence is common without addressing underlying instability through rehabilitation or surgery.

**Question 3.** **What are the clinical manifestations of patellar dislocation?**

**DeepSeek-R1 response:**

The clinical manifestations of patellar dislocation can be categorized as follows:

**1. Symptoms**

- **Acute Pain**: Sudden, severe pain during the dislocation, often localized to the anterior knee.
- **Instability Sensation**: Feeling the knee "give way" or a "popping" sensation during the dislocation.
- **Inability to Bear Weight**: Difficulty walking or standing on the affected leg.
- **Limited Motion**: Inability to fully flex or extend the knee due to pain or mechanical blockage.

**2. Physical Signs**

- **Visible Deformity**: The patella is visibly displaced laterally (most common), creating an abnormal knee contour.
- **Swelling and Effusion**: Rapid onset due to hemarthrosis (bleeding into the joint) or synovial inflammation.
- **Tenderness**: Palpable tenderness over the medial retinaculum or medial femoral condyle.
- **Apprehension Sign**: Anxiety or resistance when attempting to push the patella laterally (positive apprehension test).
- **Ecchymosis**: Bruising around the knee, particularly if associated with soft-tissue or bone injury.

**3. Functional Impairments**

- **Locking or Catching**: Inability to fully straighten the knee if the patella remains subluxated or if there is an osteochondral fragment.
- **Recurrent Instability**: History of prior dislocations or subluxations (common in patients with ligamentous laxity or anatomical abnormalities).

**4. Associated Injuries**

- **Medial Patellofemoral Ligament (MPFL) Tear**: The primary stabilizer of the patella, often injured during dislocation.
- **Osteochondral Fractures**: Bone or cartilage damage from the patella striking the lateral femoral condyle.
- **Chondral Lesions**: Cartilage injury, leading to crepitus (grinding sensation) during knee movement.

**5. Chronic Manifestations**

- **Persistent Instability**: Recurrent dislocations due to weakened ligaments or predisposing factors (e.g., trochlear dysplasia, patella alta).
- **Patellofemoral Arthritis**: Long-term complication from cartilage damage.

**Clinical Evaluation**

- **Imaging**: X-rays may show lateral displacement, osteochondral fragments, or predisposing anatomy. MRI can assess soft-tissue damage (MPFL, cartilage).

Prompt reduction (manual or spontaneous) is typical, but evaluation for underlying structural issues is critical to prevent recurrence. Management often involves immobilization, physical therapy, or surgical repair in cases of recurrent instability or significant tissue damage.

**Question 4. How is patellar dislocation diagnosed?**

**DeepSeek-R1 response:**

Diagnosing a patellar dislocation involves a comprehensive approach combining clinical evaluation and imaging studies. Here's a structured breakdown:

1. **Patient History**

- **Mechanism of Injury**: Sudden twist, direct blow, or knee hyperextension during sports or a fall.
- **Symptoms**: Acute pain, feeling the kneecap "pop" or shift laterally, immediate swelling, and possible difficulty bearing weight.
- **Recurrence**: History of prior dislocations suggests chronic instability.
- **Post-Reduction**: Spontaneous relocation may occur, leaving residual pain and swelling.

2. **Physical Examination**

- **Visual Inspection**: Observe for deformity, swelling (effusion), or bruising. A laterally displaced patella may be visible in acute cases.
- **Palpation**: Tenderness along the medial retinaculum or medial patellar facet.
- **Apprehension Test**: Lateral pressure on the patella elicits pain or resistance, indicating instability.
- **Patellar Mobility**: Assess lateral/medial glide; excessive movement suggests ligamentous laxity.
- **Range of Motion**: Limited due to pain or effusion; crepitus may indicate cartilage damage.

3. **Imaging Studies**

- **X-rays**:
  - **AP/Lateral Views**: Evaluate patellar position, fractures (e.g., medial patellar avulsion), or trochlear dysplasia.
  - **Sunrise/Merchant View**: Assess patellofemoral alignment; lateral displacement or tilt may be seen.
- **MRI**: Identifies soft tissue injuries (e.g., medial patellofemoral ligament tear, cartilage lesions, bone bruising).
- **CT Scan**: Analyzes bony anatomy (trochlear groove depth, patellar tilt) in recurrent cases or surgical planning.

4. **Differential Diagnosis**

- Exclude ACL/meniscus tears, femoral/tibial fractures, or patellar tendon rupture via specific tests (e.g., Lachman, McMurray).

5. **Special Considerations**

- **Recurrent Dislocations**: Assess for predisposing factors (e.g., ligament laxity, malalignment).
- **Pediatric Patients**: Evaluate for osteochondral fractures or growth plate involvement.

Conclusion

Diagnosis hinges on correlating history (trauma, recurrent instability) with physical findings (apprehension, tenderness) and imaging evidence of dislocation or sequelae (bone bruising, ligament tears). Early identification of associated injuries guides appropriate management.

**Question 5. How is patellar dislocation treated?**

**DeepSeek-R1 response:**

**Treatment of Patellar Dislocation**
Patellar dislocation, where the kneecap shifts out of its groove, is managed through a structured approach depending on whether it is a first-time or recurrent injury, and the presence of complications.

**1. Acute Management**

- **Reduction**:
  - **Manual Reduction**: A healthcare professional gently repositions the patella. Sedation or analgesics may be used to ease discomfort.
  - **Spontaneous Reduction**: The patella may slip back naturally during knee extension.
- **Immobilization**:
  - A knee brace or splint is applied to keep the knee straight for 2–4 weeks, allowing soft tissue healing.
- **Imaging**:
  - **X-rays/MRI** to detect fractures (e.g., osteochondral fractures) or ligament damage (e.g., medial patellofemoral ligament (MPFL) tear).

**2. Conservative Treatment** (First-time dislocations without complications)

- **RICE Protocol**:
  - **Rest**, **Ice**, **Compression**, **Elevation** to reduce swelling and pain.
- **Medications**:
  - NSAIDs (e.g., ibuprofen) for pain and inflammation.
- **Physical Therapy**:
  - Focus on strengthening the quadriceps, hamstrings, and hip stabilizers.
  - Gait training and proprioceptive exercises to improve knee stability.
- **Bracing/Taping**:
  - Patellar-stabilizing braces or kinesiology tape during activities to prevent recurrence.

**3. Surgical Intervention** (For recurrent dislocations or complications)

- **Indications**:
  - Osteochondral fractures, loose bodies, MPFL tears, or failed conservative treatment.
- **Procedures**:
  - **MPFL Reconstruction**: Rebuilding the torn ligament to stabilize the patella.
  - **Tibial Tubercle Transfer**: Realigning the patellar tendon attachment.
  - **Lateral Release**: Cutting tight lateral ligaments to reduce lateral pull.
  - **Arthroscopy**: Removal of loose cartilage/bone fragments.

**4. Rehabilitation and Prevention**

- **Activity Modification**: Avoiding high-risk movements (e.g., pivoting sports) until strength improves.
- **Long-term Bracing**: For athletes or physically active individuals.
- **Follow-up**: Monitoring for recurrent instability or degenerative changes.

**Key Considerations**

- **First-time Dislocations**: Typically managed conservatively unless complications arise.
- **Recurrent Cases**: Higher likelihood of requiring surgery to address anatomical abnormalities.
- **Complications**: Osteoarthritis, chronic instability, or cartilage damage with repeated dislocations.

This approach balances immediate care, rehabilitation, and prevention to restore function and reduce recurrence risk.

**Question 6. How effective is conservative treatment of patellar dislocation?**

**DeepSeek-R1 response:**

The effectiveness of conservative treatment for patellar dislocation depends on factors such as the type of dislocation (acute vs. habitual), structural abnormalities, and patient-specific characteristics. Below is a synthesis of evidence from the provided search results:

**Key Findings on Conservative Treatment Effectiveness**

1. **Comparable Long-Term Outcomes to Surgery**
   A 2012 meta-analysis comparing surgical and conservative treatments for acute patellar dislocation found no significant differences in recurrence rates, long-term knee function (Kujala scores), or reoperation rates between the two approaches. However, surgical treatment showed **better short-term functional recovery** (2–5 years post-treatment) in Kujala scores compared to conservative management.^1,2^
2. **Recurrence Rates**
   The recurrence rate after conservative treatment is similar to surgery in the long term. For example, the meta-analysis reported a risk ratio (RR) of 0.54 for recurrence with surgery versus conservative treatment, but the confidence interval (95% CI: 0.28–1.07) indicated no statistically significant difference.^1^
3. **First-Line Approach for Acute Cases**
   Conservative treatment is typically recommended for **first-time acute patellar dislocations without major structural damage** (e.g., medial patellofemoral ligament [MPFL] tears or cartilage fractures). It involves immobilization with a brace or cast for 4–6 weeks, followed by physical therapy to strengthen the quadriceps and improve patellar tracking.^3,4^
4. **Limitations in Habitual or Structural Cases**
   For patients with **habitual dislocations or anatomical abnormalities** (e.g., high Q-angle, shallow trochlear groove), conservative treatment is less effective. Surgical intervention is often required to correct underlying issues such as ligament reconstruction or bony realignment.^3,5^
5. **Post-Treatment Monitoring**
   After conservative management, close monitoring for vascular complications (e.g., via ankle-brachial index or Doppler ultrasound) is critical, as knee dislocation can damage the popliteal artery.^4^

**When to Choose Conservative Treatment**

- **Acute, first-time dislocations** with minimal soft-tissue damage.
- **Low-risk patients** without predisposing anatomical abnormalities.
- **Non-athletes or individuals with low physical demands**.

**Advantages and Disadvantages**

| **Advantages** | **Disadvantages** |
| --- | --- |
| Avoids surgical risks (infection, scarring) | Slower functional recovery compared to surgery^1^ |
| Lower initial cost | Higher recurrence risk in cases with structural defects^3^ |
| Suitable for uncomplicated cases | Requires strict adherence to rehabilitation protocols |

**Recommendations from Studies**

- **Meta-analysis (2012)**: Conservative and surgical treatments yield comparable long-term outcomes, but surgery may offer faster recovery in the short term.^1^
- **Clinical Guidelines**: Conservative management is preferred for primary dislocations, while surgery is reserved for recurrent cases or significant anatomical abnormalities.^2,3^

**Conclusion**

Conservative treatment is **effective for acute, uncomplicated patellar dislocations**, with outcomes similar to surgery in the long term. However, patients with recurrent dislocations or structural defects may require surgical correction to reduce recurrence and improve stability. Individualized assessment of anatomical factors and patient activity levels is critical for optimal outcomes.

**Question 7. What are the risks associated with surgical treatment of patellar dislocation?**

**DeepSeek-R1 response:**

The surgical treatment of patellar dislocation, while often effective, carries several risks and potential complications. These can be categorized as follows:

**General Surgical Risks**

1. **Infection**: Risk of superficial or deep joint infection (septic arthritis), requiring antibiotics or additional surgery.
2. **Bleeding/Blood Clots**: Hematoma formation, deep vein thrombosis (DVT), or pulmonary embolism (PE).
3. **Anesthesia Complications**: Adverse reactions, respiratory issues, or cardiovascular events.
4. **Scarring**: Cosmetic concerns or internal adhesions leading to stiffness.

**Procedure-Specific Risks**

1. **Nerve/Vessel Damage**:
   - **Numbness/Weakness**: Injury to the infrapatellar branch of the saphenous nerve, causing numbness near the incision.
   - **Vascular Injury**: Rare but serious compromise to blood flow.
2. **Recurrent Instability**: Failure to correct alignment, leading to redislocation (5–30% risk depending on technique).
3. **Stiffness/Limited Motion**: Due to postoperative scarring or arthrofibrosis, often requiring aggressive rehabilitation.
4. **Patellar Fracture**: Especially in procedures involving bone manipulation (e.g., tibial tubercle osteotomy).
5. **Overcorrection/Medial Instability**: Over-tightening medial structures, causing the patella to shift medially.
6. **Hardware Issues**: Pain or irritation from screws/implants, sometimes necessitating removal.
7. **Graft Failure**: Stretching or rupture of MPFL grafts (autograft/allograft).
8. **Cartilage Damage**: Iatrogenic injury during surgery or altered tracking leading to chondral lesions.

**Long-Term Complications**

1. **Post-Traumatic Arthritis**: Accelerated cartilage wear due to altered joint mechanics.
2. **Chronic Pain**: Persistent anterior knee pain or complex regional pain syndrome (CRPS).
3. **Delayed Healing/Non-Union**: In osteotomies, bone may fail to heal properly.

**Rehabilitation Challenges**

- Poor adherence to physical therapy, leading to suboptimal outcomes.
- Prolonged recovery (6–12 months) with potential for delayed return to sports.

**Factors Influencing Risk**

- **Patient-Specific**: Age, obesity, smoking, or preexisting conditions (e.g., connective tissue disorders).
- **Surgical Technique**: Lateral release alone has higher recurrence rates vs. MPFL reconstruction with osteotomy.

**Conclusion**

While surgery can stabilize the patella effectively, risks must be weighed against benefits. Individualized approaches, surgeon experience, and postoperative compliance are critical to minimizing complications. Patients should discuss these risks thoroughly with their orthopedic surgeon.

**Question 8. What are the surgical methods for patellar dislocation?**

**DeepSeek-R1 response:**

The surgical treatment of patellar dislocation is tailored to the underlying anatomical abnormalities and patient-specific factors. Based on current research and clinical practices, the primary surgical methods include:

**1. Medial Patellofemoral Ligament (MPFL) Reconstruction**

This is the **gold standard** for recurrent patellar dislocation. The procedure involves replacing the damaged or lax MPFL with a graft (autograft, allograft, or synthetic material) to restore medial stability. Key advancements include:

- **Anatomic reconstruction**: Grafts are fixed at the femoral and patellar insertion sites using bone tunnels or absorbable screws to replicate natural ligament tension.^6,7^
- **Combined procedures**: Often paired with lateral retinacular release or tibial tubercle osteotomy (TTO) for complex cases with trochlear dysplasia or abnormal TT-TG distance (>20 mm).^7,8^
- **Outcomes**: Postoperative recurrence rates are as low as 0–14%, with significant improvements in Lysholm and Kujala scores.^6,7^

**2. Tibial Tubercle Osteotomy (TTO)**

Used to correct patellar tracking by adjusting the tibial tubercle position. Common techniques include:

- **Elmslie-Trillat osteotomy**: Medializes and/or derotates the tibial tubercle to reduce the Q-angle and TT-TG distance, improving patellofemoral alignment.^7,8^
- **Fulkerson osteotomy**: Combines medialization and anteriorization of the tubercle for patients with patellofemoral arthritis.^8^
- **Applications**: Particularly effective for patients with trochlear dysplasia or excessive lateral patellar tilt.^8^

**3. Trochleoplasty**

Addresses femoral trochlear dysplasia, a major risk factor for recurrent dislocation. Techniques include:

- **Sulcus-deepening osteotomy**: Reshapes the shallow trochlear groove to enhance patellar engagement.^6^
- **Lateral facet elevation**: Corrects hypoplastic lateral femoral condyles.^6^
- **Indications**: Recommended for severe Dejour type B–D trochlear dysplasia.^8^

**4. Lateral Retinacular Release**

A less invasive procedure involving the release of tight lateral structures to reduce lateral patellar pull. However, it is **no longer recommended as a standalone treatment** due to high recurrence rates (up to 23%) and is typically combined with MPFL reconstruction.^3,6^

**5. Osteotomy for Anatomical Abnormalities**

- **Patellar osteotomy**: Reshapes the patella in cases of Cyrano-type patella (elongated inferior pole) to improve sagittal alignment and reduce instability.^9^
- **Femoral osteotomy**: Corrects rotational malalignment (e.g., excessive femoral anteversion) contributing to patellar instability.^3^

**6. Combined Procedures**

For multifactorial instability, hybrid approaches are often employed:

- **MPFL reconstruction + TTO + trochleoplasty**: Addresses ligamentous laxity, bony malalignment, and trochlear dysplasia simultaneously.^8^
- **Dynamic medial stabilization**: Augments MPFL reconstruction with adductor magnus tendon transfer for enhanced medial pull.^9^

**Postoperative Management**

Rehabilitation focuses on early mobilization, quadriceps strengthening, and proprioceptive training. Functional outcomes (e.g., Tegner scores) may not fully return to pre-injury levels, but pain and instability are significantly reduced.^7,8^

**Key Considerations**

- **Individualized treatment**: Surgical plans must account for factors like age, activity level, and anatomical variations (e.g., patella alta, Q-angle).^3,6^
- **Imaging**: Preoperative CT/MRI is critical to assess TT-TG distance, patellar tilt, and trochlear morphology.^6,10^
- **Complications**: Risks include graft failure, patellar fracture, and arthrofibrosis, but these are minimized with precise surgical techniques.^7^

For further details, refer to the cited studies on MPFL reconstruction,^6,7^ trochleoplasty, ^6^and combined osteotomies.^8^

**Question 9. How should rehabilitation exercises be conducted post-surgically for patellar dislocation?**

**DeepSeek-R1 response:**

Rehabilitation following surgical correction of a patellar dislocation should be structured in phases, focusing on restoring function while protecting healing tissues. Here's a comprehensive approach:

**Phase 1: Acute Phase (0–2 Weeks Post-Op)**

**Goals:** Control pain/swelling, protect surgical repair, initiate gentle mobility, and prevent muscle atrophy.

- **Protection:** Use a knee brace or immobilizer (locked in extension for weight-bearing if required). Gradual weight-bearing per surgeon’s guidelines (may require crutches).
- **Modalities:**
  - RICE (Rest, Ice, Compression, Elevation).
  - Electrical stimulation for quadriceps activation if voluntary contraction is limited.
- **Exercises:**
  - Isometric quadriceps sets ("quad squeezes") and gluteal sets.
  - Ankle pumps (to improve circulation).
  - Straight-leg raises (supine, no resistance) once pain allows.
  - Gentle patellar mobilizations (if cleared by surgeon).

**Phase 2: Subacute Phase (2–6 Weeks Post-Op)**

**Goals:** Restore range of motion (ROM), improve strength, and initiate proprioception.

- **ROM:** Progress to 0–90° flexion by week 4, then full ROM by week 6. Use heel slides, wall slides, and seated assisted flexion.
- **Strengthening:**
  - Mini squats (0–30° knee flexion), step-ups (2–4 inches), and leg presses (limited ROM).
  - Terminal knee extensions (TKEs) with resistance bands.
  - Hip-focused exercises (clamshells, side-lying leg lifts) to address gluteal strength.
- **Proprioception/Balance:** Single-leg stands, weight shifts, and balance board drills (seated initially).

**Phase 3: Strengthening Phase (6–12 Weeks Post-Op)**

**Goals:** Build strength, endurance, and dynamic stability.

- **Closed Kinetic Chain Exercises:**
  - Squats (progress depth to 60–90°), lunges, and lateral step-ups.
  - Leg press with increased ROM and resistance.
- **Open Kinetic Chain Exercises:**
  - Short-arc quad extensions (avoid terminal 30° extension initially if MPFL repaired).
- **Balance/Agility:**
  - Single-leg balance on unstable surfaces (foam, BOSU), cone drills, and lateral shuffles.
- **Cardio:** Stationary bike (low resistance), swimming (flutter kicks avoided early), or elliptical.

**Phase 4: Advanced Training (3–6 Months Post-Op)**

**Goals:** Sport-specific drills, power, and return to activity.

- **Plyometrics:**
  - Box jumps, jump landings (focus on knee alignment), and agility ladder drills.
- **Running Program:**
  - Gradual progression from jogging to sprinting (ensure proper mechanics).
- **Criteria for Return to Sport:**
  - Full ROM, >85% quad strength vs. contralateral limb, successful hop tests, and no pain/swelling.

**Key Considerations**

1. **VMO Activation:** Emphasize exercises like step-downs, TKEs, and biofeedback to enhance medial quadriceps recruitment.
2. **Avoid Valgus Stress:** Modify exercises to prevent knee collapsing inward (e.g., monitor form during squats/lunges).
3. **Bracing/Taping:** May be used temporarily during higher-risk activities.
4. **Education:** Address biomechanics (e.g., hip/core strengthening, footwear).
5. **Multidisciplinary Care:** Regular communication between PT, surgeon, and patient to adjust protocols based on healing.

**Precautions**

- Avoid deep flexion (e.g., deep squats) until cleared.
- Monitor for persistent swelling, crepitus, or instability.
- Progress based on functional milestones, not just time.

**Conclusion**

Rehabilitation must be individualized, criteria-driven, and supervised by a physical therapist. Early phases prioritize protection and activation, while later phases focus on strength and sport-specific demands. Consistent monitoring ensures safe progression and reduces reinjury risk.

**Question 10. How soon can I return to sports after patellar dislocation surgery?**

**DeepSeek-R1 response:**

Returning to sports after patellar dislocation surgery is a gradual process that depends on the type of surgery, individual healing, and adherence to rehabilitation. Here's a general timeline and key considerations:

**Phases of Recovery**

1. **Early Post-Op (Weeks 1–6):**
   - **Immobilization:** Use of a brace or cast to protect the knee.
   - **Pain Management and Swelling Control:** Focus on reducing inflammation.
   - **Initial Physical Therapy (PT):** Gentle range-of-motion exercises and quadriceps activation.
2. **Intermediate Phase (Weeks 6–12):**
   - **Progressive PT:** Increase mobility, strength, and weight-bearing activities.
   - **Low-Impact Exercise:** Stationary biking or swimming may begin.
3. **Strengthening Phase (Months 3–6):**
   - **Strength and Stability Training:** Emphasis on quadriceps, hamstrings, and hip muscles.
   - **Balance/Proprioception Work:** To restore knee control.
4. **Sport-Specific Training (Months 4–6+):**
   - **Functional Drills:** Agility, jumping, and cutting exercises.
   - **Gradual Return:** Start with non-contact practice, progressing to full activity.

**Return to Sports Timeline**

- **Non-Contact Sports (e.g., running, swimming):** ~4–6 months.
- **High-Demand Sports (e.g., soccer, basketball):** ~6–9 months, sometimes longer.

**Key Factors Influencing Timeline**

- **Type of Surgery:** MPFL reconstruction, osteotomy, or cartilage repair may extend recovery.
- **Rehab Adherence:** Strict compliance with PT accelerates progress.
- **Sport Demands:** Cutting, pivoting, or contact sports require longer rehab.
- **Healing Milestones:** Full range of motion, ≥85% strength in the affected leg vs. the healthy leg, and no pain/swelling.

**Medical Clearance**

- **Functional Tests:** Hop tests, single-leg squats, and sport-specific drills.
- **Imaging:** MRI or X-ray may be used to confirm healing.
- **Surgeon/PT Approval:** Final decision is individualized.

**Risks of Early Return**

- Re-dislocation, cartilage damage, or chronic instability if the knee isn’t fully rehabilitated.

**Summary**

Most athletes return to sports **4–9 months post-surgery**, but timelines vary. Always follow your surgeon’s and physical therapist’s guidance to ensure a safe return. Patience and thorough rehab are critical to minimizing reinjury risk.

**References**

1.	Tian W, Wang F ming, Huang J, et al. Meta analysis on the treatment of acute patellar dislocation by surgical versus conservative treatment. *Modern Rehabilitation*. 2012;(44):8228-8234.

2.	Tian G, Yang G, Zuo L, Li F, Wang F. Conservative versus repair of medial patellofemoral ligament for the treatment of patients with acute primary patellar dislocations: A systematic review and meta-analysis. *J Orthop Surg (Hong Kong)*. 2020;28(2):2309499020932375. doi:10.1177/2309499020932375

3.	Patellar dislocation - Baidu Wenku. Accessed April 24, 2025. <https://wenku.baidu.com/view/6487ac8f312b3169a551a427.html?_wkts_=1745480587740>.

4.	Knee dislocation - China Emergency Medicine Network. Accessed April 24, 2025. <http://www.cem.org.cn/magazine/index.php/default/content/index/id/11563>.

5.	Inferior patellar branch of the descending genicular artery, pronunciation, translation, English example sentence, English dictionary. Accessed April 24, 2025.<http://www.dictall.com/indu/093/09260326937.htm>.

6.	Research Advances in Surgical Treatment of Recurrent Patellar Dislocation. *Advances in Clinical Medicine*. 2024;14(04):379. doi:10.12677/acm.2024.1441033

7.	Zhang H, Hong L, Geng X, Wang X, Feng H. [Reconstruction of medial patellofemoral ligament for recurrent patellar dislocation]. *Zhongguo Xiu Fu Chong Jian Wai Ke Za Zhi*. 2011;25(8):925-930.

8.	The journal industry - All Journals - Collects all the magazines in the world - Disseminates academic achievements - Professional journal search - Journal informatization - Academic search. Accessed April 24, 2025.	<https://yyws.alljournals.cn/relate_search.aspx?pcid=A9DB1C13C87CE289EA38239A9433C9DC&cid=527A01A248DACB72&aid=829257168E98218A8642C8B7A233440D&language=0&ctl=14&etl=0>.

9.	Yang K, Prescher A, Hildebrand F, Weber CD. Anatomical and clinical significance of the Cyrano long-nosed patella in combination with patellofemoral instability: a case report and review of literature. *Journal of Medical Case Reports*. 2025;19(1):186. doi:10.1186/s13256-025-05235-w

10.	Missed Transient Patellar Dislocation. Accessed April 24, 2025. <http://www.360doc.com/content/22/1108/21/47509206_1055134906.shtml>.
